# Pioneering the implementation of a precision oncology strategy in Portugal: the Precision Oncology Platform trial

**DOI:** 10.2340/1651-226X.2023.33322

**Published:** 2024-06-23

**Authors:** Beatrice Mainoli, Joana Assis, José Dinis, Rui Henrique, Júlio Oliveira

**Affiliations:** a Clinical Research Unit, Research Center of IPO Porto (CI-IPOP)/RISE@CI-IPOP (Health Research Network), Portuguese Oncology Institute of Porto (IPO Porto)/Porto Comprehensive Cancer Center Raquel Seruca (Porto.CCC Raquel Seruca), R. Dr. António Bernardino de Almeida, Porto, Portugal; b Department of Medical Oncology, Portuguese Oncology Institute of Porto (IPO-Porto)/Porto Comprehensive Cancer Center Raquel Seruca (Porto.CCC Raquel Seruca), R. Dr. António Bernardino de Almeida, Porto, Portugal; c Department of Pathology and Cancer Biology & Epigenetics Group, Research Center of IPO Porto (CI-IPOP)/CI-IPOP @RISE (Health Research Network), Portuguese Oncology Institute of Porto (IPO Porto)/Porto Comprehensive Cancer Centre Raquel Seruca (Porto.CCC Raquel Seruca), R. Dr. António Bernardino de Almeida, Porto, Portugal; d Department of Pathology and Molecular Immunology, ICBAS-School of Medicine & Biomedical Sciences, University of Porto (ICBAS-UP), Porto, Portugal; e Experimental Pathology and Therapeutics Group, Research Center (CI-IPOP)/RISE@CI-IPOP (Health Research Network), Portuguese Oncology Institute of Porto (IPO-Porto)/Porto Comprehensive Cancer Center Raquel Seruca (Porto.CCC Raquel Seruca), R. Dr. António Bernardino de Almeida, Porto, Portugal

**Keywords:** Precision oncology, targeted treatment, DRUP-like Clinical Trials, Precision Oncology Platform (POP) trial

## Abstract

**Background and purpose:**

The Precision Oncology Platform (POP) trial represents the effort of the Portuguese Oncology Institute of Porto (IPO Porto) for joining other leading European institutions in both ‘Personalised Cancer Medicine for all EU citizens’ (PCM4EU), and ‘PRecisIon Cancer MEdicine RepurpOsing SystEm Using Pragmatic Clinical Trials’ (PRIME-ROSE) consortia, enabling the development of the Portuguese version of the Drug Rediscovery Protocol (DRUP)-like Clinical Trial (DLCT), based on the experience of the DRUP trial developed in The Netherlands.

**Patients/material and methods:**

The POP trial is a phase II, pragmatic multicentric, non-randomised, open-label study, designed entirely like the other DLCTs. Its primary objective is to describe anti-tumour activity of targeted anticancer drugs in patients with advanced malignancies harbouring actionable molecular alterations. The primary endpoint is disease control rate (DCR). Secondary endpoints encompass treatment-related grade ≥3 adverse events, objective response rate (ORR), duration of response (DOR), progression-free survival (PFS), and overall survival (OS). Exploratory objectives will assess biomarkers, resource use and costs, and patient-reported outcome measures (PROMs).

**Interpretation:**

The POP trial will offer access to innovative treatments for patients without further therapeutic options and provide evidence on efficacy and safety of molecularly-guided treatments. Methodologically, it represents a pioneer approach in Portugal, including a pay-for-performance model embedded in the clinical trial. The POP trial represents a unique opportunity to integrate clinical research within cancer care, pursuing an evidence-based precision oncology strategy, and facilitating its rational and cost-effective implementation into the Portuguese healthcare system.

## Introduction

The implementation of Personalised Cancer Medicine (PCM) represents a transformative shift, promising to overhaul traditional approaches to cancer care. This paradigm leverages targeted therapies to benefit patients whose tumours exhibit specific molecular traits. Increasing knowledge on cancer hallmarks has precipitated the development of innovative treatments, typically relying on tumour molecular profiling and predictive biomarkers to guide therapeutic allocations. Targeted drugs pipeline is rapidly expanding, with accelerated clinical development and regulatory approvals. However, granting of agnostic indications remains constrained, particularly within Europe, where concomitant reimbursement issues often limit access to novel treatments.

Dissemination of PCM poses significant challenges. Widespread expertise in interpretation of complex genomic reports, constituting the baseline for rational use of targeted agents, is lacking. Often, such targeted treatments are marketed drugs prescribed off-label or investigational agents available only through clinical trials. Off-label prescription raises concerns due to unproven efficacy in many cases, as well as effectiveness and safety data on its use are generally not systematically recorded and analysed in routine clinical practice. Unequal access is also a concern because of high cost of new targeted agents and reimbursement issues, contributing to heterogeneity in PCM implementation. Expanded/managed access and compassionate use programmes are very limited, not offering sustainable treatment options, and often lack systematic collection of evidence. Based on the experience of the *Drug Rediscovery Protocol* (DRUP) trial developed in The Netherlands, several European institutions have decided to implement *DRUP-like Clinical Trials (DLCTs)*. Nordic countries were the first to open parallel national protocols (IMPRESS-Norway, ProTarget, FINPROVE, MEGALIT). Subsequently, several European partners established a network that will facilitate the exchange of data between the trials, and joined the consortia *Personalised Cancer Medicine for all EU citizens –* PCM4EU [1] and the *PRecisIon Cancer MEdicine RepurpOsing SystEm Using Pragmatic Clinical Trials* (PRIME-ROSE) [2].

The main objective of DLCTs is to assess the efficacy and toxicity of commercially available targeted anticancer drugs for treatment of advanced cancer disclosing potentially actionable alterations. These projects are expected to generate clinical evidence and address the effectiveness of PCM strategies, promoting evidence-based treatment interventions that improve outcomes in cancer care. Through harmonisation and collaboration, the consortia will enable expedited evidence generation for rare mutations/tumour types, due to simultaneous data collection. Additionally, these consortia will cooperate with regulators, policymakers, payers, healthcare providers, and patient advocacy groups to implement evidence based PCM in routine practice, facilitating the rational and cost-effective implementation of the results into the healthcare systems.

The *Precision Oncology Platform* (POP) trial represents the effort of the Portuguese Oncology Institute of Porto (IPO Porto) in joining other leading European institutions in both PCM4EU and PRIME-ROSE consortia, enabling the development of the Portuguese version of DLCTs. IPO Porto is a national reference centre in clinical research, which has been strengthened since the creation of the first Early Phase Clinical Trials Unit dedicated to oncology in Portugal. It has been in charge of implementing precision oncology strategies in our comprehensive cancer centre. Considering the increasing accessibility to sequencing technologies, establishing an institutional multidisciplinary Molecular Tumour Board (MTB) was mandatory for interpretation of molecular results and to guide their rational use for clinical decision-making. Concomitantly, IPO Porto pioneered the establishment of a molecular screening programme, designed as a research project, to allow the implementation of a precision oncology strategy. Indeed, IPO Porto’s Precision Oncology Program (POP-IPOP) was developed as a single-site, tumour type-agnostic, prospective observational study, aiming to evaluate the feasibility of using molecular profile-based evidence to support individualised cancer therapy for patients with advanced/refractory, rare or hard-to-treat cancers. Overall, this strategy complies with the recommendation for research centres to perform multigene sequencing as part of the mission to accelerate cancer research and drug development, providing patients access to innovation and prospectively collecting data on the use of next-generation sequencing (NGS) in clinical practice [3] One of the main goals of POP-IPOP, through the MTB, is to accelerate patients’ inclusion in clinical trials, and reducing the allocation to off-label treatments. Since its implementation in 2021, IPO Porto screening programme has included more than 400 patients, and its regional expansion is planned in the short term. Therefore, the POP trial will be essential to provide additional treatment options for these patients, but also for patients from other hospitals, reaching nationwide coverage.

## Methods

### Study design and POP trial state-of-the-art

The POP trial is a phase II, pragmatic multicentric, non-randomised, open-label clinical trial designed to evaluate the efficacy and safety of off-label use of commercially available targeted anticancer drugs. The design of this trial is entirely similar to other DLCTs, described elsewhere [4–6], with just a few local specificities.

The study incorporates three subsequent stages, opening according to the success of current cohorts.

The POP trial protocol has already been presented and discussed with both Pharma companies, represented by the Portuguese Association of Pharmaceutical Industry (APIFARMA) and the Portuguese Regulatory Authority (INFARMED). It will be submitted for formal approval after negotiation and conclusion of the scientific advice process.

IPO Porto is the sponsor of this trial. Additional recruiting Portuguese centres are expected to be opened.

### Study objectives and endpoints

The trial’s primary objective is to describe anti-tumour activity of targeted anticancer drugs among patients with advanced malignancies harbouring actionable molecular alterations. The primary endpoint is disease control rate (DCR), measured by the proportion of patients achieving complete response (CR), partial response (PR), or stable disease (SD) 16 weeks post-treatment initiation.

Secondary objectives include further assessing the efficacy and safety of tested drugs, evaluate patient-reported outcome measures (PROMs), and examine health-related quality of life. Secondary endpoints encompass the following: proportion of patients with treatment-related grade ≥3 and serious adverse events; objective response rate (ORR), duration of response (DOR), progression-free survival (PFS), and overall survival (OS).

Exploratory objectives will include immune and metabolic tumour responses, genomic/transcriptomic analysis, circulating tumour DNA assessment or microbiome evaluation. Additionally, description of resource use and related costs, together with PROMs will be performed.

### Patient population and cohort assignment

Adult and paediatric patients with advanced or metastatic solid tumours or haematological malignancies will be enrolled. They must show disease progression to standard treatments or have no acceptable treatment options. Enrolment is contingent upon availability of tumour genomic or protein expression test results, demonstrating a potentially actionable mutation and availability of study drugs.

Key inclusion criteria include: adequate organ function, measurable disease, and an Eastern Cooperative Oncology Group (ECOG) performance status of 0–2.

The trial assigns patients to specific cohorts defined by their tumour type, molecular profile, and targeted agent.

eDrug-specific study manuals describe drug-specific inclusion and exclusion criteria, dosing, toxicity management, projected risk-benefit assessments and treatment schedules.

Negotiations with several pharmaceutical companies, undertaken through the Portuguese Pharma Association [7], are in their final steps, allowing for an equitable and transparent approach under a common memorandum of understanding.

### Statistical considerations

As every other DLCT, the POP trial involves a Simon-like two-stage ‘admissible’ design [8,9] for assessing targeted anticancer drug efficacy across multiple cohorts. Each cohort, defined by tumour type, molecular variant, and treatment, starts with eight participants in stage I. The absence of responses prompts early cohort cessation, while any positive response leads to a stage II expansion to 24 participants. This methodology balances the need for a minimal sample size against the trial’s statistical integrity. Response rates below 10% signal clinical disinterest, whereas 30% or above advocate for further investigation (stage III). The selected monitoring rule yields an 85% power with a 7.8% alpha error rate. If stage II yields at least 5 patient responses, the cohort advances to stage III for validation of clinical benefit rate with 80%–90% statistical power (based on clinical benefit rate observed in stage II). The power and sample size calculation will be performed individually for each cohort proceeding to stage III. The POP trial supports data exchange with parallel DLCTs to ensure a robust sample size enabling reliable analysis of all cohorts.

### Collateral research

This trial will enable further research, as collateral studies are envisaged, especially for translational purposes and health technology assessment (HTA). Namely, collaboration with several institutional research groups will be crucial for biomarkers analysis. Participants’ biological material will be stored at IPO Porto biobank.

Finally, analyses of performance-based risk-sharing agreements (RSA) embedded in clinical research are planned, aiming to methodologically characterise this innovative strategy, its application, and economic impact in the Portuguese healthcare system.

## Discussion

A DLCT implementation represents the best opportunity to integrate clinical research within routine cancer care, pursuing a PCM strategy in Portugal. We emphasise the relevance and innovation of the POP trial based on three main aspects: it offers access to innovative treatments for patients without further treatment options; it provides an evidence-generation platform to inform on efficacy and safety of molecularly-guided treatments, and it allows for inclusion of a pay-for-performance model within the clinical trial design.

Integrating PCM and new technologies into healthcare systems constitutes a challenge and public health policies are required to ensure its rational use. Few countries have implemented structured national policies in Europe [10–13], even if some have developed either some legal framework or national plans [14–19]. In Portugal, a coordinated national strategy for PCM implementation is lacking [20]. The European Federation of Pharmaceutical Industries and Associations (EFPIA) recently developed policy recommendations to improve cancer care through broader access to quality biomarker testing [21]. Indeed, a limitation of POP-trial is that molecular screening is not included within the trial, so patients may only be referred if a potentially actionable alteration was already identified. As the access to comprehensive genome sequencing is heterogeneous, referral might also be heterogeneous, showing that additional efforts are needed to develop a PCM ecosystem in Portugal.

Challenges of PCM are also particularly relevant for HTA, especially concerning agnostic indications. These trials have been termed ‘histology-inclusive’, as they maintain the tissue-agnostic orientation of precision oncology, without losing the relevance of histologic categorisation [22]. DLCTs have an additional cutting-edge feature: stage III constitutes a pay-for-performance model: after the first 4 months of treatment, supplied by the pharmaceutical companies, drugs are covered, only if clinical benefit is confirmed. This constitutes an ‘implementation device’ for PCM and aims to expand access to drugs besides generating clinical evidence; such an approach also allows for reframing of healthcare as a ‘learning system’, re-centred on research and simultaneously providing inputs to implementation of healthcare policies in PCM [22].

PCM value assessment framework still needs to build consensus among multiple perspectives, fostering procedures and measures of value aspects [23]. The POP trial’s findings will contribute to a dialogue that extends beyond the oncology domain [24], addressing broader questions around drug repurposing, dynamic treatment guidelines, and the economic viability of targeted therapeutics, to guarantee sustainability of healthcare systems. We believe in a patient-centred approach, promoting access to cutting-edge technologies, integrated into an evidence-based context, supporting evidence generation strategies, both from clinical trials and real-world evidence (RWE).

From a value-based perspective, RSA strategies are considered promising tools, enabling outcome-based coverage/reimbursement [25,26]. The POP trial, by including Stage III, represents, to the best of our knowledge, a pioneering approach in Portugal. Indeed, establishing an RSA embedded in a clinical trial represents an innovative strategy, even from a methodological point of view. In our perspective, clinical research should approximate to earlier HTA, addressing the current gaps in access to medicines, and such type of innovative features of RSA included in clinical trials may represent a possible strategy in this regard. This vision is consistent with the new HTA Regulation [27] and EUnetHTA21 initiative [28] aiming at harmonisation of HTA across Europe and improving equity and access to innovative medicines.

Future trends are expected to evolve towards integrating patient-centred clinical research in a tailored approach. Thus, we believe that the POP trial will become the catalyst for implementing PCM in Portugal, aiming to produce evidence and accelerate drug development, allowing rational and sustainable use of molecular-guided therapies.

## Data Availability

Not applicable – no data presented.
